# *GSTP1* Methylation and Protein Expression in Prostate Cancer: Diagnostic Implications

**DOI:** 10.1155/2016/4358292

**Published:** 2016-08-10

**Authors:** Filippo Martignano, Giorgia Gurioli, Samanta Salvi, Daniele Calistri, Matteo Costantini, Roberta Gunelli, Ugo De Giorgi, Flavia Foca, Valentina Casadio

**Affiliations:** ^1^Biosciences Laboratory, Istituto Scientifico Romagnolo per lo Studio e la Cura dei Tumori (IRST) IRCCS, Via P. Maroncelli 40, 47014 Meldola, Italy; ^2^University of Florence, 50121 Florence, Italy; ^3^Pathology Unit, Morgagni-Pierantoni Hospital, 47121 Forlì, Italy; ^4^Department of Urology, Morgagni-Pierantoni Hospital, 47121 Forlì, Italy; ^5^Department of Medical Oncology, Istituto Scientifico Romagnolo per lo Studio e la Cura dei Tumori (IRST) IRCCS, Via P. Maroncelli 40, 47014 Meldola, Italy; ^6^Unit of Biostatistics and Clinical Trials, Istituto Scientifico Romagnolo per lo Studio e la Cura dei Tumori (IRST) IRCCS, Via P. Maroncelli 40, 47014 Meldola, Italy

## Abstract

*GSTP1* belongs to the GSTs family, a group of enzymes involved in detoxification of exogenous substances and it also plays an important role in cell cycle regulation. Its dysregulation correlates with a large variety of tumors, in particular with prostate cancer. We investigated* GSTP1* methylation status with methylation specific PCR (MS-PCR) in prostate cancer (PCa) and in benign tissue of 56 prostatectomies. We also performed immunohistochemistry (IHC) so as to correlate gene methylation with gene silencing.* GSTP1* appears methylated in PCa and not in healthy tissue; IHC confirmed that methylation leads to protein underexpression (*p* < 0.001). GSTP1 is highly expressed in basal cell layer and luminal cells in benign glands while in prostatic intraepithelial neoplasia (PIN) it stains only basal cell layer, whereas PCa glands are completely negative. We demonstrated that methylation leads to underexpression of GSTP1. The progressive loss of GSTP1 expression from healthy glands to PIN and to PCa glands underlines its involvement in early carcinogenesis.

## 1. Introduction

*GSTP1* belongs to the glutathione S-transferases (GSTs) family, enzymes that catalyze the detoxification of endogenous and exogenous substances conjugating them with glutathione (GSH) [1].

These enzymes interact with several factors (such as regulatory kinases) thus modulating signaling pathways involved in cell proliferation, differentiation, and apoptosis.

Therefore, GST plays an important role in cancer cell proliferation and death thanks to its cytoprotective and regulatory functions [2].

Alterations in epigenetic regulation mechanisms, such as promoter hypermethylation, are often involved in tumor development, progression, and recurrence [3–5].

*GSTP1* methylation is frequently associated with tumor development or poor prognosis in a wide range of tumors such as neuroblastoma [6], hepatocellular carcinoma [7], endometrial [8], breast [9], and prostate cancers (PCa) [10]. It is involved in the early process of carcinogenesis in PCa [11, 12] and its methylation has been largely investigated: about 70–80% of PCa cases are methylated, while benign prostatic hyperplasias are normally hypomethylated [13, 14].

*GSTP1* methylation has been proposed by many research papers as an epigenetic marker for early prostate cancer diagnosis [15] and it is certainly the most widely studied epigenetic marker in prostate cancer in the last decade [12].

Loss of GSTP1 expression, as a consequence of gene methylation, seems to be one of the first events to cause a preneoplastic phenotype to develop into a malignant phenotype [11]. Some literature data have shown a correlation between promoter hypermethylation and reduction of GSTP1 expression in PCa [16, 17]. However, these data still need to be confirmed.

In our study, we aimed to verify and confirm the inverse correlation between methylation and GSTP1 expression in different stages of tumorigenesis and better understand the role of this marker in early carcinogenesis.

We chose to perform methylation and expression analyses on prostatectomies. With respect to biopsied tissue, the larger tissue area from prostatectomy allowed easier isolation of the PCa area and the adjacent healthy tissue for better methylation analyses and evaluation of GSTP1 expression.

Prostatectomy also facilitates detection of preneoplastic lesions that are considered a transition point between healthy phenotype and full-blown malignant phenotype [18], such as prostatic intraepithelial neoplasia (PIN).

As Kang et al. demonstrated that* GSTP1* methylation is heterogeneous in PIN [19], it could be interesting to investigate GSTP1 expression in this type of tissue to better understand its role in tumor development.

Finally GSTP1 expression could potentially represent a good histological marker in substitution of or in addition to AMACR, p63, or cytokeratin [20–22], already used in clinical practice, so as to improve PCa detection when diagnosis is ambiguous due to the presence of cancer mimics such as PIN.

We investigated the GSTP1 gene methylation status using methylation specific PCR (MS-PCR) and assessed methylation correlation with gene silencing through immunohistochemistry (IHC) in cancer and healthy paraffin embedded tissues obtained from prostatectomy. To further confirm the role of GSTP1 in the early stages of prostatic neoplasia, we also evaluated protein expression in preneoplastic lesions.

## 2. Materials and Methods

### 2.1. Case Series

We collected 56 formalin fixed paraffin embedded (FFPE) tissue samples from patients submitted to prostatectomy between 2012 and 2014. For each sample we collected both PCa tissue and the corresponding adjacent healthy tissue from prostatectomy. From 16 of these patients we also collected FFPE tissue from the biopsies obtained before surgery.

Before taking part in the study, all patients signed the written informed consent reviewed and approved by the local Ethics Committee. All samples were retrieved from the Archives of the Pathology Unit at the Morgagni-Pierantoni Hospital in Forlì. Case series details such as age, Gleason score, pathological stage, and PSA value are shown in Table 1.

### 2.2. Macrodissection and DNA Isolation

Cancer and adjacent healthy tissues from prostatectomy were selected by a pathologist and macrodissected on the basis of hematoxylin-eosin sections. Healthy prostatic tissue was macrodissected at a distance of 7 mm from the tumor sample. DNA was extracted using QIAamp DNA FFPE Tissue Kit (Qiagen, Milan, Italy), according to the manufacturer's instructions, and quantified by spectrophotometry (NanoDrop ND-1000, Celbio, Milan, Italy).

### 2.3. Methylation Specific PCR

DNA was converted with sodium bisulphite using EZ DNA Methylation-Gold*™* Kit (Zymo Research Corporation, Irvine, USA). The reactions were performed using 100 ng of DNA extracted from LNCaP cell line (methylated control) and from the peripheral blood of a healthy volunteer (unmethylated control), with 150 ng of DNA extracted from FFPE tissues. We performed real-time PCR using SYBR-GREEN master mix (Biorad, Milan, Italy) and primers specific for bisulphite-converted DNA. We tested the DNA quality by Actin B amplification, using 1 *μ*L of bisulphite-converted DNA. Real-time PCR for* Actin B* was performed using Rotor-Gene 3000 (Diatech Pharmacogenetics, Jesi, Italy) under the following conditions: 95°C for 5 minutes and then 40 cycles at 94°C for 30 seconds, at 62°C for 60 seconds, and then at 72°C for 60 seconds. We then assessed PCR product specificity with melt curve analysis and set the Ct threshold at 0.02. Primer sequences for* Actin B* are shown:

 5′-TGGTGATGGAGGAGGTTTAGTAAGT-3′,
 5′-AACCAATAAAACCTACTCCTCCCTTAA-3′,

as described elsewhere [21]. After that, real-time PCR for* GSTP1* was performed with 2 *μ*L of bisulphite-converted DNA samples under the following conditions: 95°C for 3 minutes and then 40 cycles at 94°C for 30 seconds, at 56°C for 60 seconds, and at 72°C for 60 seconds. We included the methylated control, the unmethylated control, and a negative control with sterile water in all reactions. All reactions were performed in duplicate and independent experiments were performed. The specificity of the reaction was verified by melting curves (melting curves and amplification plots are shown in Supplementary Figure S1, in Supplementary Material available online at http://dx.doi.org/10.1155/2016/4358292).* GSTP1* primers for methylated and unmethylated sequences are shown as follows:

 
*For methylated  sequences,*
   5′-TATCGTGGTTTATTTTTTAGTTCGA-3′,
   3′-ATAAAAAAATTCGAATCTCTCCGA-5′.
 
*For unmethylated sequences,*
   5′-TATTGTGGTTTATTTTTTAGTTTGA-3′,
   3′-ATAAAAAAATTCAAATCTCTCCAAA-5′.

Amplicon location is shown in Supplementary Figure  S2.

### 2.4. Immunohistochemistry

FFPE tissue samples were cut into 5 *μ*m sections, deparaffinized, and rehydrated.

Antigen unmasking was performed with citrate buffer (pH 6) at 98.5°C for 30 minutes.

The samples were first incubated with H_2_O_2_ to block endogenous peroxidase activity and then incubated with Anti-GST3/GST pi antibody [EPR8263] rabbit monoclonal antibody (Abcam, Cambridge, UK) for 1 hour (diluted 1 : 500), with secondary biotinylated antibody (Dako REAL*™* Detection Systems LSAB*™*+, K5001 HRP/DAB+, Rabbit/Mouse) and with HRP-conjugated streptavidin (Dako REAL*™* Detection Systems LSAB*™*+, K5001 HRP/DAB+, Rabbit/Mouse).

The samples were stained with diaminobenzidine (DAB) (Dako REAL*™* Detection Systems LSAB*™*+, K5001 HRP/DAB+, Rabbit/Mouse) as a peroxidase substrate and counterstained with hematoxylin.

All samples were analyzed by a pathologist for GSTP1 expression. Tissues presenting more than 20% of positive cells with an intensity of at least 1+ were considered positive for GSTP1 expression.

### 2.5. Statistical Analysis

Frequency tables with percentages were performed for categorical variables while continuous variables were presented using median and range. The relationship between* GSTP1* methylation and its expression in tissue was analyzed using Fisher exact test.

*p* values < 0.05 were considered statistically significant. Statistical analyses were performed using STATA/MP version 10.1 (StataCorp LP, USA) statistical software.

## 3. Results

Analysis of the PCa samples and the corresponding adjacent healthy prostatic tissue showed that* GSTP1* is methylated in 51 tumor samples (91.1%) and in 3 adjacent healthy tumor samples (5.4%) as shown in Table 2.

All 56 prostatectomy samples showed GSTP1 expression in adjacent healthy tissue irrespective of methylation pattern.

All 51 (100.0%) cases methylated for* GSTP1* in PCa tissue showed no expression. Out of the 5 unmethylated PCa tissues, only 2 (40.0%) expressed GSTP1.

We observed an inverse association between methylation and expression of GSTP1 (*p* < 0.001) in the overall series. A similar inverse relation was observed in cancer tissues (*p* = 0.006).

In healthy tissues almost all samples were unmethylated (94.6%) with a high GSTP1 expression; however all the 3 methylated samples had a corresponding gene expression and no samples with lack of expression were found, so it was impossible to obtain a statistically significant association (*p* = 1.000).

All the 18 cases that have a low Gleason score (≤6) and all the 7 T2a tumors are unmethylated in healthy tissue and methylated in PCa with a consequent GSTP1 expression in healthy tissue and loss of expression in PCa.

**Figure 1:**
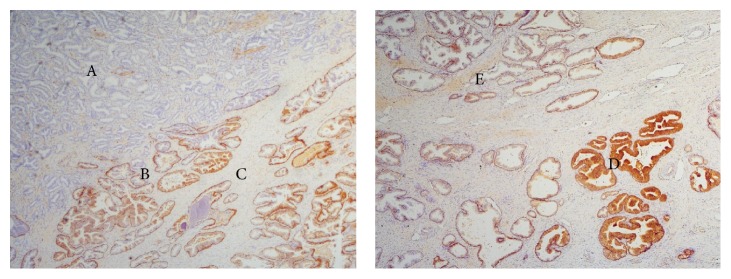
Typical GSTP1 staining pattern in prostatectomy: benign glands strongly positive (A), PIN basal cell layer positive with negative luminal cells (B), and PCa completely negative (C). Heterogeneous GSTP1 staining in benign glands in prostatectomy: strong positivity (D) and weaker positivity (E).

**Figure 2:**
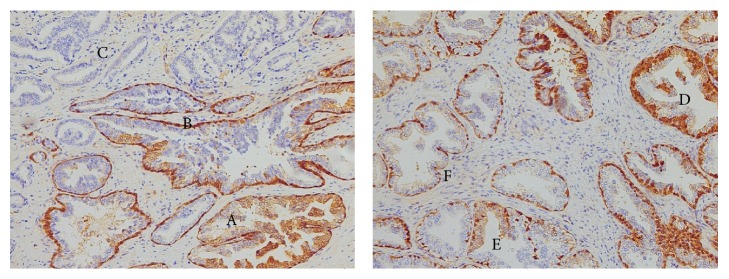
Detail of typical GSTP1 staining pattern in prostatectomy: benign glands strongly positive (A), PIN basal cell layer positive with negative luminal cells (B), and PCa completely negative (C). Detail of heterogeneous GSTP1 staining in benign glands in prostatectomy: strong positivity in basal cell layer and luminal cells (D), intermediate positivity in luminal cells and strong positivity in basal cell layer (E), and strong positivity in basal cell layer and negative luminal cells (F).

GSTP1 expression in benign tissue is heterogeneous: it strongly stains healthy gland basal cell layer, while it frequently shows a weaker positivity or even absence of staining in luminal cells (Figures 1 and 2).

PIN glands generally show a remarkably weaker staining in luminal cells compared to healthy glands and often present absence of staining. On the other hand, the basal cell layer shows good positivity (Figures 1 and 2).

Malignant glands do not express GSTP1 at all, and they are completely negative due to the lack of the basal cell layer (Figures 1 and 2).

All the biopsies analyzed belong to patients whose* GSTP1* status in prostatectomies was unmethylated in healthy tissue and methylated in PCa with a consequent GSTP1 expression in healthy tissue and loss of expression in PCa.

**Figure 3:**
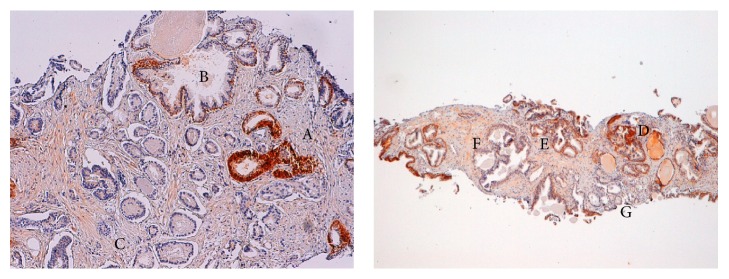
Typical GSTP1 staining pattern in biopsy: benign glands strongly positive (A), positive basal cell layer with negative luminal cells in PIN (B), and PCa completely negative (C). Dishomogeneous GSTP1 staining in benign glands in biopsy: strong (D), intermediate (E), and weak (F). PCa is completely negative (G).

GSTP1 expression in biopsies reflects the results obtained on prostatectomies (Figure 3).

## 4. Discussion

*GSTP1* seems to be involved in different tumor types, due to its role in detoxification of exogenous substances and regulation of cell cycle, and its overexpression is often associated with drug resistance [2, 23]. Dysregulation of* GSTP1* methylation is frequent in different tumor types [6–9]. Moreover, with regard to prostate cancer, extensive literature data have demonstrated its hypermethylation [10–15]. More than twenty years ago some papers demonstrating that* GSTP1* methylation is an early event in prostatic carcinogenesis appeared in the literature [24, 25]; in the following years a number of papers and reviews have been published on the role of* GSTP1* methylation as a potential diagnostic marker [26, 27]. Despite the fact that* GSTP1* methylation is certainly a good marker for prostate cancer, it is not currently used in clinical practice and further validation studies are needed. The present study aimed to validate the role of* GSTP1* methylation and to correlate it with protein expression.

In line with previous studies, we found* GSTP1* methylation in 91.1% of tumor tissues and in 5.4% of adjacent healthy tissues. We thus confirmed that* GSTP1* methylation is a cancer specific biomarker and we enforced the concept that* GSTP1* could be an early diagnostic marker.

The analysis of GSTP1 expression by IHC confirmed that hypermethylation correlates with underexpression in malignant glands in almost every sample, whereas it is strongly expressed in healthy tissues (100.0%).

It is worth mentioning that while* GSTP1* hypermethylation often results in gene silencing in PCa tissue, an unmethylated status of* GSTP1* with a loss of expression was observed in 3 cases of PCa. This suggests that GSTP1 suppression may be due to other regulation mechanisms, such as miRNAs or other epigenetic factors.

Another important point is that GSTP1 presents heterogeneous expression in benign tissue: it strongly stains healthy gland basal cell layer, while it frequently shows a weaker positivity or even an absence of staining in luminal cells (Figures 1 and 2).

In particular, PIN glands generally showed a weaker staining in luminal cells compared to healthy glands and often absence of staining, while the basal cell layer shows good positivity (Figures 1 and 2). On the contrary, malignant glands do not express GSTP1 at all and result as completely negative, also due to the lack of basal cell layer (Figures 1 and 2).

It is noteworthy that* GSTP1* appears to be methylated and silenced also in patients with low grade (≤6) and low stage (T2a), confirming its involvement in early carcinogenesis and suggesting that GSTP1 alterations (methylation or expression) may be considered as useful early diagnostic markers, so as to avoid unnecessary rebiopsies, as recently demonstrated by Zelic et al. [28].

Subsequently, we performed immunohistochemistry analyses on 16 prostate needle biopsies in order to understand whether GSTP1 staining could be helpful for the histological evaluation of core biopsies for diagnostic purpose. Thanks to its capacity for staining in basal cell layer, we hypothesized that GSTP1 could be used to discriminate benign prostatic hyperplasia and PIN (which maintains basal cell staining uniformity) from PCa (which lacks a basal layer).

In biopsies, GSTP1 has a staining pattern comparable to that obtained on prostatectomies (Figure 3). It is still able to stain healthy glands and basal cell layer in PIN but, unfortunately, it does not prove satisfactorily reliable due to its nonhomogeneous staining in noncancerous tissues.

In line with our results, Kang et al. found dishomogeneous results for PIN methylation status of* GSTP1*, ascertaining that about half of the samples were methylated [19]. Indeed, PIN is a borderline tissue that undergoes a transition process from a normal expression to a loss of expression of GSTP1 in PCa.

In conclusion, although GSTP1 does not seem to be useful for histological evaluation of core biopsies, as previously demonstrated [29], its behavior in the various stages of tumor development is interesting and should be better investigated. In healthy glands GSTP1 shows a stronger positivity than PIN, where luminal cells partially or totally lose GSTP1 expression, whereas in PCa a total negativity is shown.

The progressive loss of GSTP1 expression may correlate with the progressive transition from a benign phenotype to PCa.

## 5. Conclusions

We confirmed that* GSTP1* methylation is an epigenetic event strongly related to PCa. Methylation analysis could be helpful to reveal PCa even in patients with low grade and low stage tumors; moreover, expression analysis further demonstrated that* GSTP1* methylation leads to gene silencing in PCa tissues.

However, GSTP1 expression did not prove a reliable marker for histological biopsy evaluation due to the high variability in preneoplastic lesions.

## Supplementary Material

Supplementary Figure S1: The results obtained by Real Time PCR (methylated and unmethylated sequences of GSTP1) are reported. Supplementary Figure S2: A picture showing primers location within GSTP1 gene is reported.

## Figures and Tables

**Table 1:** Case series.

		*n* (%)
All cases		56 (100.0)

Age, years	≤70	46 (82.1)
	>70	10 (17.9)

Gleason score	≤6	18 (32.1)
	>6	38 (67.9)

Pathological stage	T2a	7 (12.5)
	T2b	0 (0.0)
	T2c	25 (44.6)
	T3a	21 (37.5)
	T3b	3 (5.4)

Median PSA level, ng/mL [range]		6.4 [2.65–29.52]

**Table 2:** Relationship between GSTP1 methylation and expression.

	Total (*n* = 112)		*p *value	PCa tissues (*n* = 56)		*p*value	Adjacent healthy tissues (*n* = 56)		*p* value
	Met. *n* (%)	Unmet. *n* (%)		Met. *n* (%)	Unmet. *n* (%)		Met. *n* (%)	Unmet. *n* (%)	
Total	54	58		51	5		3	53	

Expression	3 (5.6)	55 (94.8)	<0.001	0 (0.0)	2 (40.0)	0.006	3 (100.0)	53 (100.0)	1.000
No expression	51 (94.4)	3 (5.2)		51 (100.0)	3 (60.0)		0 (0.0)	0 (0.0)	

## References

[1] Laborde E. (2010). Glutathione transferases as mediators of signaling pathways involved in cell proliferation and cell death. *Cell Death and Differentiation*.

[2] Singh S. (2015). Cytoprotective and regulatory functions of glutathione S-transferases in cancer cell proliferation and cell death. *Cancer Chemotherapy and Pharmacology*.

[3] Sarkar S., Horn G., Moulton K. (2013). Cancer development, progression, and therapy: an epigenetic overview. *International Journal of Molecular Sciences*.

[4] Vecchio L., Seke Etet P. F., Kipanyula M. J., Krampera M., Nwabo Kamdje A. H. (2013). Importance of epigenetic changes in cancer etiology, pathogenesis, clinical profiling, and treatment: what can be learned from hematologic malignancies?. *Biochimica et Biophysica Acta (BBA)—Reviews on Cancer*.

[5] Casadio V., Molinari C., Calistri D. (2013). DNA methylation profiles as predictors of recurrence in non muscle invasive bladder cancer: an MS-MLPA approach. *Journal of Experimental & Clinical Cancer Research*.

[6] Gumy-Pause F., Pardo B., Khoshbeen-Boudal M. (2012). GSTP1 hypermethylation is associated with reduced protein expression, aggressive disease and prognosis in neuroblastoma. *Genes Chromosomes and Cancer*.

[7] Li Q. F., Li Q. Y., Gao A. R., Shi Q. F. (2015). Correlation between promoter methylation in the *GSTP1* gene and hepatocellular carcinoma development: a meta-analysis. *Genetics and Molecular Research*.

[8] Fiolka R., Zubor P., Janusicova V. (2013). Promoter hypermethylation of the tumor-suppressor genes RASSF1A, GSTP1 and CDH1 in endometrial cancer. *Oncology Reports*.

[9] Fang C., Wei X.-M., Zeng X.-T., Wang F.-B., Weng H., Long X. (2015). Aberrant GSTP1 promoter methylation is associated with increased risk and advanced stage of breast cancer: a meta-analysis of 19 case-control studies. *BMC Cancer*.

[10] Goering W., Kloth M., Schulz W. A. (2012). DNA methylation changes in prostate cancer. *Methods in Molecular Biology*.

[11] Schnekenburger M., Karius T., Diederich M. (2014). Regulation of epigenetic traits of the glutathione S-transferase P1 gene: from detoxification toward cancer prevention and diagnosis. *Frontiers in Pharmacology*.

[12] Costa V. L., Henrique R., Jerónimo C. (2007). Epigenetic markers for molecular detection of prostate cancer. *Disease Markers*.

[13] Andrés G., Ashour N., Sánchez-Chapado M., Ropero S., Angulo J. C. (2013). The study of DNA methylation in urological cancer: present and future. *Actas Urologicas Espanolas*.

[14] Jiang D., Shen Y., Dai D. (2014). Meta-analyses of methylation markers for prostate cancer. *Tumor Biology*.

[15] Henrique R., Jerónimo C. (2004). Molecular detection of prostate cancer: a role for GSTP1 hypermethylation. *European Urology*.

[16] Zhang W., Jiao H., Zhang X. (2015). Correlation between the expression of DNMT1, and GSTP1 and APC, and the methylation status of GSTP1 and APC in association with their clinical significance in prostate cancer. *Molecular Medicine Reports*.

[17] Lin X., Tascilar M., Lee W.-H. (2001). GSTP1 CpG island hypermethylation is responsible for the absence of GSTP1 expression in human prostate cancer cells. *The American Journal of Pathology*.

[18] Bostwick D. G., Liu L., Brawer M. K., Qian J. (2004). High-grade prostatic intraepithelial neoplasia. *Reviews in Urology*.

[19] Kang G. H., Lee S., Lee H. J., Hwang K. S. (2004). Aberrant CpG island hypermethylation of multiple genes in prostate cancer and prostatic intraepithelial neoplasia. *The Journal of Pathology*.

[20] Epstein J. I., Egevad L., Humphrey P. A. (2014). Best practices recommendations in the application of immunohistochemistry in the prostate: report from the International Society of Urologic Pathology Consensus Conference. *The American Journal of Surgical Pathology*.

[21] Singh V., Manu V., Malik A., Dutta V., Mani N. S., Patrikar S. (2014). Diagnostic utility of p63 and *α*-methyl acyl Co A racemase in resolving suspicious foci in prostatic needle biopsy and transurethral resection of prostate specimens. *Journal of Cancer Research and Therapeutics*.

[22] Kuroda N. (2014). Application of combined immunohistochemical panel of AMACR(P504S)/p63 cocktail, cytokeratin 5 and D2-40 to atypical glands in prostatic needle biopsy. *Malaysian Journal of Pathology*.

[23] Sawers L., Ferguson M. J., Ihrig B. R. (2014). Glutathione S-transferase P1 (GSTP1) directly influences platinum drug chemosensitivity in ovarian tumour cell lines. *British Journal of Cancer*.

[24] Lee W.-H., Morton R. A., Epstein J. I. (1994). Cytidine methylation of regulatory sequences near the π-class glutathione S-transferase gene accompanies human prostatic carcinogenesis. *Proceedings of the National Academy of Sciences of the United States of America*.

[25] Lee W.-H., Isaacs W. B., Bova G. S., Nelson W. G. (1997). CG island methylation changes near the GSTP1 gene in prostatic carcinoma cells detected using the polymerase chain reaction: a new prostate cancer biomarker. *Cancer Epidemiology, Biomarkers & Prevention*.

[26] Van Neste L., Herman J. G., Otto G., Bigley J. W., Epstein J. I., Van Criekinge W. (2012). The epigenetic promise for prostate cancer diagnosis. *The Prostate*.

[27] Wu T., Giovannucci E., Welge J., Mallick P., Tang W.-Y., Ho S.-M. (2011). Measurement of GSTP1 promoter methylation in body fluids may complement PSA screening: a meta-analysis. *British Journal of Cancer*.

[28] Zelic R., Fiano V., Zugna D. (2016). Global hypomethylation (LINE-1) and gene-specific hypermethylation (GSTP1) on initial negative prostate biopsy as markers of prostate cancer on a rebiopsy. *Clinical Cancer Research*.

[29] Sailer V., Eberhard H. L. K., Stephan C. (2015). Glutathione S-transferase-pi protein expression in prostate cancer-not always a useful diagnostic tool. *Histopathology*.

