# Supplementary material for: GSTP1 Methylation and Protein Expression in Prostate Cancer: Diagnostic Implications
Source: Dis Markers. 2016 Aug 10;2016:4358292. doi: 10.1155/2016/4358292 (PMC4995330; doi:10.1155/2016/4358292)
Supplement: Supplementary file 1 — Supplementary Figure S1: The results obtained by Real Time PCR (methylated and unmethylated sequences of GSTP1) are reported. Supplementary Figure S2: A picture showing primers location within GSTP1 gene is reported. [file 4358292.f1.pdf]

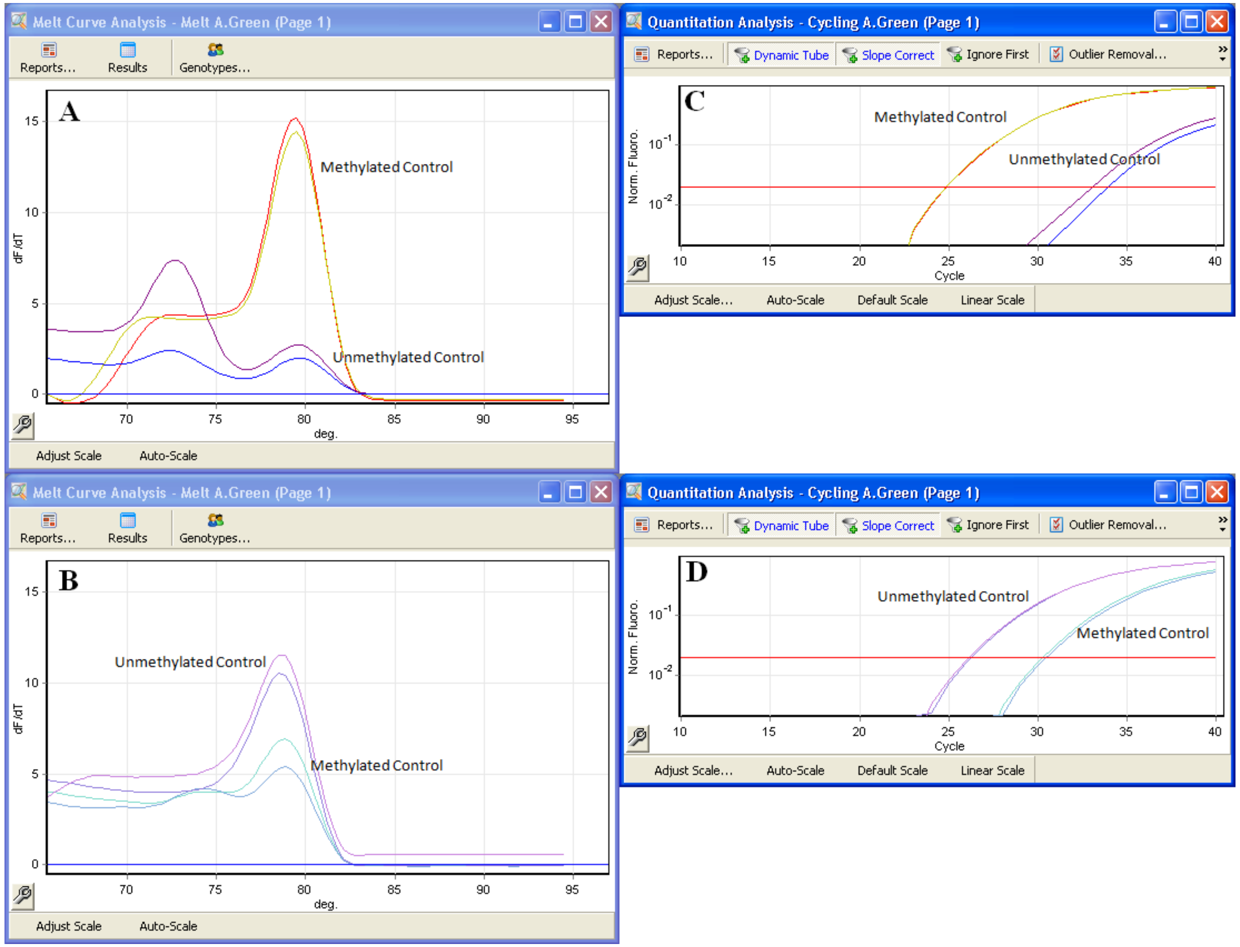

Supplementary Figure S1

Melting curves for A) methylated and B) unmethylated reactions. Amplification plot for C) methylated and D) unmethylated reactions

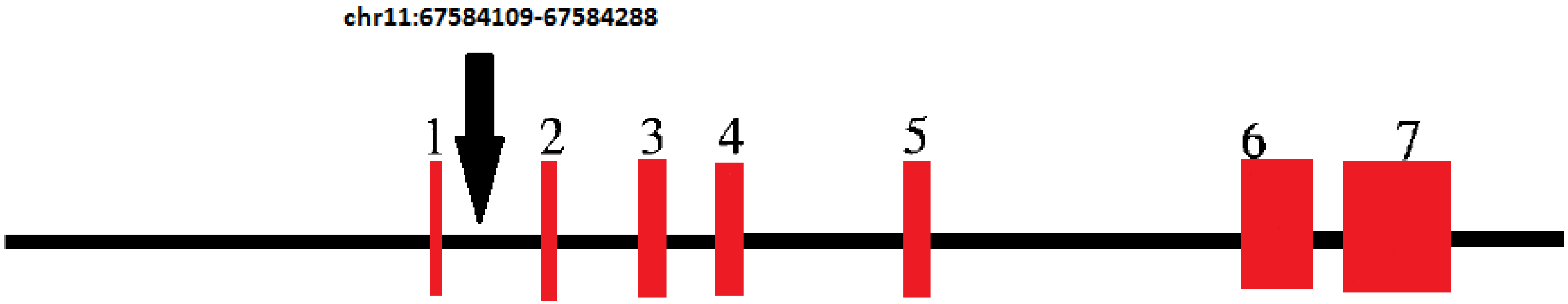

Supplementary Figure S2

GSTP1 amplicon is located in chromosome 11 and starts at 67584109 and ends at 67584288 position. The exons are represented in red.
